# Sub-optimal delivery of intermittent preventive treatment for malaria in pregnancy in Nigeria: influence of provider factors

**DOI:** 10.1186/1475-2875-11-317

**Published:** 2012-09-07

**Authors:** Chima A Onoka, Obinna E Onwujekwe, Kara Hanson, Benjamin S Uzochukwu

**Affiliations:** 1 Health Policy Research Group, College of Medicine, University of Nigeria, Enugu Campus, Enugu, Nigeria; 2 London School of Hygiene and Tropical Medicine, Keppel Street, London, UK; 3 Department of Community Medicine, College of Medicine, University of Nigeria, Enugu, Nigeria; 4 Department of Pharmacology and Therapeutics, College of Medicine, University of Nigeria, Enugu, Nigeria

**Keywords:** Malaria, Intermittent preventive treatment, Pregnancy, Provider factors, Nigeria, Supply

## Abstract

**Background:**

The level of access to intermittent preventive treatment for malaria in pregnancy (IPTp) in Nigeria is still low despite relatively high antenatal care coverage in the study area. This paper presents information on provider factors that affect the delivery of IPTp in Nigeria.

**Methods:**

Data were collected from heads of maternal health units of 28 public and six private health facilities offering antenatal care (ANC) services in two districts in Enugu State, south-east Nigeria. Provider knowledge of guidelines for IPTp was assessed with regard to four components: the drug used for IPTp, time of first dose administration, of second dose administration, and the strategy for sulphadoxine-pyrimethamine (SP) administration (directly observed treatment, DOT). Provider practices regarding IPTp and facility-related factors that may explain observations such as availability of SP and water were also examined.

**Results:**

Only five (14.7%) of all 34 providers had correct knowledge of all four recommendations for provision of IPTp. None of them was a private provider. DOT strategy was practiced in only one and six private and public providers respectively. Overall, 22 providers supplied women with SP in the facility and women were allowed to take it at home. The most common reason for doing so amongst public providers was that women were required to come for antenatal care on empty stomachs to enhance the validity of manual fundal height estimation. Two private providers did not think it was necessary to use the DOT strategy because they assumed that women would take their drugs at home. Availability of SP and water in the facility, and concerns about side effects were not considered impediments to delivery of IPTp.

**Conclusion:**

There was low level of knowledge of the guidelines for implementation of IPTp by all providers, especially those in the private sector. This had negative effects such as non-practice of DOT strategy by most of the providers, which can lead to low levels of adherence to IPTp and ineffectiveness of IPTp. Capacity development and regular supportive supervisory visits by programme managers could help improve the provision of IPTp.

## Background

Nigeria adopted the intermittent preventive treatment for malaria in pregnancy (IPTp) strategy in 2001 [1]. Although studies in Nigeria show the efficacy of IPTp in preventing anaemia in pregnancy among Nigerian women [2-4], there is still low coverage of the intervention in Nigeria. The most recent demographic and health survey (DHS) in Nigeria revealed that both first and second dose coverage remain low, being 8.0% and 4.6% respectively in Nigeria, and 9.9% and 5.4% in south-east Nigeria [5]. A recent study [6] reported values of 13.7% and 7.3% for first and second doses, respectively.

IPTp using sulphadoxine-pyrimethamine (SP) is given to pregnant women during antenatal care visits on at least two occasions following quickening; a dose during the second and during the third trimesters of pregnancy under direct observation [7]. Antenatal care (ANC) is provided by skilled birth attendants, including midwives, doctors or nurses, who have been educated and sufficiently trained to provide care for women during pregnancy, delivery and the six-week period following pregnancy [8]. Although data about the health facility type used by women for ANC are unavailable, 25.3% and 48.6% of live birth deliveries in south-east Nigeria occur in public and private facilities respectively, with the remainder occurring mainly at home [5]. While 58% of pregnant women in Nigeria receive ANC from a skilled birth attendant, the figure is 87% for the south-east region of the country [5]. However, attendance at antenatal care does not guarantee effective delivery of IPTp as favourable provider-side factors need to be in place as well [9].

Since antenatal clinics serve as the usual entry point for IPTp implementation [10], the nature of service provision in the clinics as well as attendance by pregnant women is key to optimal IPTp coverage. Facility and policy-related factors were reported as being more serious impediments to IPTp coverage in Tanzania than the timing of ANC attendance [11]. The poor level of knowledge of guidelines for IPTp delivery amongst health workers in Malawi negatively affected IPTp delivery [12]. Health workers have also been found to offer all women IPTp (including first trimester clients) during their first clinic visit [13]. In some cases, health workers are confused about the appropriate timing of first dose, and spacing between doses of SP, resulting in low coverage [12]. In Malawi some health workers were of the opinion that SP should not be taken on an empty stomach, which reduced the delivery of SP under direct observation [12]. Unavailability of clean water in Malawi, drug stock-outs in Kenya, Tanzania, Uganda and Zambia, and staff shortages in antenatal clinics in Uganda have also negatively impacted coverage levels [9].

Studies in Nigeria have reported low knowledge of IPTp guidelines amongst a sample of all cadres of health care providers [14], and poor experience of directly-observed treatment (DOT) strategy among ANC attendees with 36.8% of women offered IPTp taking it in the facility and only 14.3% doing so under health worker observation [15]. However, little is known about the knowledge and practices of the health providers who primarily play the role of ANC providers in health facilities.

This paper provides new information about the provider factors at ANC clinic level that affect the delivery of IPTp in Nigeria and complements the evidence generated by the demand side component of the study, which suggested that supply rather than demand side factors may be responsible for the low coverage of IPTp in Nigeria [6]. The paper provides information on health worker knowledge of IPTp policy, actual health worker practices, the underlying reasons for such practices, and other facility requirements for IPTp delivery. Such information is critical for understanding supply-side impediments to effectiveness of the IPTp policy and identifying areas for supply-side interventions for improvement of provision of IPTp.

## Methods

### Study area

The study was conducted in Enugu North and South local government areas (LGA) and Udi LGA in Enugu State, south-east Nigeria, in 2010. Udi LGA (rural) has a population of 244,852, and Enugu North and South (urban) have a combined population of 443,575 [16]. The public health care delivery system in the state is organized in three levels based on a District Health System with primary level facilities being the entry point for health care utilization. This level also serves as the primary point of delivery of ANC services. In Udi and Enugu, 17 and 11 of such public health facilities, respectively, offer ANC services. In addition, there are several private health facilities in Enugu while six major ones exist in Udi. Each health facility has an ANC/maternity unit that has responsibility for providing ANC, family planning and delivery services. An ANC/maternity unit is headed by staff of the facility with relevant skills for maternal health service delivery, and ANC clinics are held twice a week.

### Context

Malaria is highly endemic in Enugu [17]. In line with the policy of the Federal Ministry of Health (FMOH) [1], the state implements IPTp delivery. Implementation of IPTp involves use of SP for IPTp, delivery of the first dose after the 16^th^ week of pregnancy, and provision of two doses for pregnant women at least four weeks apart, and three for HIV-positive pregnant women. Health workers are not to give SP within the four-week period preceding a woman’s expected date of delivery, quite unlike the WHO regulation which allows IPTp to be offered at this time [7], and it must not be offered within the first trimester of pregnancy. The strategy also stipulates that the health providers should give SP under direct observation at the health facility. Healthcare providers that provide ANC services receive training through workshops organized by the state Ministry of Health, and other non-governmental organizations involved in malaria control. These organizations also distribute posters and leaflets that specify the guidelines for delivery of IPTp to both public and private health care facilities [18].

### Data collection

Data were collected using an in-depth interview guide and a checklist. The interview guide was used to collect data from the heads of the ANC/maternity units in all the 28 public primary health facilities offering ANC services in Enugu and Udi, and from an additional six purposely selected private hospitals (three from each district). The information obtained included health workers’ knowledge of the existence of a policy on IPTp, the procedures for delivery of IPTp, as well as the reasons underlying observed practices. In-depth interviews were recorded using a digital voice recorder. A facility checklist was also used to obtain information on availability of SP and water for ANC on the day that the facility was visited. In addition the number of ANC clients, and the skilled attendants as defined by WHO [8] that ran the ANC clinics for the week preceding the facility visit were obtained from the facility staff register to enable determination of the ratio of ANC client/skilled attendant per clinic day. The number of unskilled staff (such as community health extension workers (CHEWs) who do not receive midwifery training) providing assistance, or fully manning, a unit was obtained.

### Data analysis

Recorded interviews were transcribed following each interview. The transcripts were coded based on predetermined themes corresponding to the main categories of interest. Information given by each provider was compared with the FMOH implementation guidelines for IPTp delivery in order to determine whether the provider had correct or incorrect knowledge of any of the components, and the number of providers that knew the four components that served as criteria for assessment of knowledge. These were: correct knowledge of the drug used, time of first dose administration, of second dose administration, and knowledge of the strategy for administration (DOT). Emphasis was then laid on describing deviations from the guidelines with regard to provider knowledge and behaviour, and the facility-related factors that may help explain observations. Apart from description of providers’ behaviour, summary statistics were obtained from the data and presented based on facility characteristics, such as urban and rural location and whether they were private or public providers. Summary statistics were also obtained from the facility checklists and categorized based on the above characteristics and the data generated were related to data from the qualitative data analysis to enhance the understanding and interpretation of the study findings. The information obtained from the study was further confirmed by participants that attended a results dissemination workshop organized at the end of the study for the health care providers, as well as state Ministry of Health officials and non-governmental organizations involved in malaria control in the state.

### Ethical concerns

Approval for this study was obtained from the Research Ethics Committee of the University of Nigeria Teaching Hospital Enugu and the London School of Hygiene and Tropical Medicine. The primary health care (PHC) coordinators for the LGAs used and the heads of primary health facilities and private hospitals gave permission for the use of facilities. Written and signed consent forms were obtained from all individuals interviewed.

## Results

### Provider knowledge of IPTp policy and guidelines for delivery

Almost all providers interviewed (94.1%) knew about the existence of the national policy on IPTp (Table 1). The Table shows that of all 34 providers interviewed, most knew the correct drug for IPTp and knew that women should receive two or three doses. However, few providers knew the drug should be received in the second and third trimesters and very few knew it should be delivered under direct observation by a health worker. Only five (14.7%) of all the providers satisfied the four criteria for assessment of knowledge of the IPTp guidelines. None of them was a private provider.

**Table 1:** Knowledge of providers about IPT for malaria in pregnancy

**Variable**	**Urban**		**Rural**		**Total**
	**Public**	**Private**	**Public**	**Private**	
	**N = 11**	**N = 3**	**N = 17**	**N =**	**N = 34**
	**n**	**n**	**n**	**n**	**n(%)**
Knowledge of existence of policy on IPTp	11	2	16	3	32 (94.1)
Correct knowledge of drug used (SP)	10	3	16	3	32 (94.1)
Correct knowledge of number of doses (2/3)	8	1	14	3	26 (76.5)
Know a dose should be given in 2^nd^ trimester	7	2	13	2	24 (70.6)
Know a dose should be given in 3^rd^ trimester	4	2	7	0	13 (38.2)
Knowledge of recommended approach of giving IPTp (DOT)	8	1	7	1	17 (50.0)
Respondents that know all the guidelines	2	0	3	0	5

Although most respondents knew the correct number of doses that should be given, the description of when they should be given (timing) varied significantly. Of the four private providers with correct knowledge of number of doses, one stated that the first dose is *“given soon after quickening* (16 weeks) *while the second is given one month after”.* Another indicated that two doses are given, as long as they are given before 36 weeks. However, they all reported that drugs are not given after 36 weeks of pregnancy.

Amongst the public providers that knew the correct number of doses, knowledge of timing of administration varied. According to one provider,*“first dose is given after 18 weeks, then second dose is a month after”* (urban provider). For another, the *“first dose is given during first trimester, the second during second trimester, and before 36 weeks you give the third dose”* (urban provider). Another provider (rural area) also stated that *“the first is given after four months of pregnancy and the second, a month later”*. Only two providers (one rural and one urban) added that a third dose is given if a patient is HIV positive. Additionally, one provider (rural) stated that three doses are given to HIV-positive women, but the dosage depends on her weight: *“Those above 50 kg take three tabs and those below take two tablets”* (rural public provider). However, she stated that the first and second doses were given in the first and second trimesters, respectively, for patients who present early, while those who present in the second trimester are given one dose in each of the second and third trimester. All the public providers reported that IPTp is not given within the last month of pregnancy.

The six public providers who had incorrect knowledge of the number of doses indicated that they provided IPTp to patients monthly. For these providers, the most common approach was to give the first dose any time after quickening and then subsequent doses *“monthly until delivery”.* None of the providers from the urban area indicated that a dose should be given in the first trimester while three providers from the rural area (all CHEWs) stated that it should.

As shown in Table 1, just half of the providers knew the correct strategy for delivery of IPTp (DOT). This figure includes providers who stated that the woman could be given the drug free or could purchase it. Twelve of the remaining 17 providers stated that the recommended approach is for the women to take the drugs home after being given the drug free of charge. One of the respondents (rural public provider) put it thus: *“a woman is given the drug free in the facility but she takes it home”.*

### Provision of IPTp in health facilities

Although about 85% of all providers reported that IPTp is provided at their facilities, the way this is done differed. According to a private provider (rural), *“we give the first dose in the first trimester, the second in the second trimester. Then we give ACT once”.* Among the public facility providers in the urban area, the deviations included that women registered at their various facilities were given “*two tablets stat and one tab*(let) *the next day”,* “*two tablets stat at 20 weeks, then one the following day at night with routine drugs and paracetamol”,*while another gives “*two tablets at five months and three at seven months when the woman is heavier”.* Similar findings were also noted from providers in public facilities in rural areas. Women were asked to take *“two tablets at night and one tablet in the morning; but if she has malaria, she takes three tablets sta*t”; *“three at once for women over 60 kg and two at once for those less than 60 kg”; “two tablets stat and one the next day”;* and *“first trimester first dose, second dose one month after; if the woman presents at second trimester, we give first dose and second dose a month after”.* In one facility, clients are given *“one tablet daily for three days to avoid reaction”.*

### Practice of directly observed treatment

Only one out of six private providers reported practicing DOT while six of the public did same (Table 2). However, for 64.7% of all providers, the woman is given the drug, which she takes home. Women receive SP free in public facilities, while those in the private facilities are billed for the drug.

**Table 2:** Factors related to provision of IPTp in health facilities

**Variable**	**Urban**		**Rural**		**Total (%)**
	**Public**	**Private**	**Public**	**Private**	
	**11**	**3**	**17**	**3**	**34**
**How IPTp is delivered**					
IPTp is offered in the facility	10	2	15	2	29 (85.3)
DOT is practiced in the facility	3	1	3	0	7 (20.6)
Woman is given drug to take home	7	1	12	2	22 (64.7)
**Where drug used for IPTp is sourced**					
Free from Ministry of Health	10	0	15	0	25 (73.5)
Purchased	0	2	0	2	4 (11.7)
**Facility occasionally has periods of drug stock-out**	6	1	4	1	12 (35.3)
**Source of drugs during stock-out**					
Direct acquisition from market for sale	1	1	1	1	4 (11.8)
Woman is given prescription to purchase on her own	5	0	3	0	8 (23.5)
**Facilities with drugs on day of interview**					
SP	11	3	15	3	32 (94.1)
ACT	8	3	16	3	30 (88.2)
Quinine	7	1	5	3	16 (47.1)
**Complaints about the drug**					
Side effect	1	2	2	1	6 (17.6)
Irregularity of drug availability	0	0	2	0	2 (5.9)

The major reason for the poor practice of DOT amongst public providers from both rural and urban areas was that most women complained of not eating before coming to the facility, and because they and the health workers considered it unsafe to take drugs on empty stomachs, they were allowed to take the drugs home. A key reason given by a health worker why women came to the hospital without eating was that *“for better palpation, women are not allowed to eat before coming. This is part of our directive* (i e, what health workers in the facility are directed to do) *and since it is risky to give drugs on empty stomach, they take it at home after eating”* (public provider – rural area). Two private facility providers saw no reason to place such an oversight on women, they believed that women knew enough of the complications of malaria and so do not have a problem with taking drugs at home. For another public provider, DOT is not practiced because *“it is not the policy of the hospital for patients to take their medication in the hospital”.* Another private provider believed that SP is *“a routine drug and is given to women along with other routine drugs”* and this is done without seeking the consent of the women or letting her know. Another private provider echoed this stating that the drug is *“given to the woman without* (her) *being told”.*

Most of the public facilities (both urban and rural) reported not having water with which to give the drug to women at the facility but believed that women had ways of overcoming this problem. Women had to buy “sachet water” if they were to take any medication in the facility (Sachet water is water provided in 500 ml capacity polybags. Most products have provisional approval by the National Agency for Food Administration and Control (NAFDAC). They are cheap ($0.06) and widely available). Some of the public providers also reported that some women used rainwater or borehole water which were free. Three private providers got water from the state-owned water cooperation but the supply was reported to be erratic. There was water for delivery of IPTp in all but one private hospital while 11 out of the 28 public facilities had water. The available water was pipe-borne in two private facilities, and sachet water in the remaining facilities that had water. Overall, only two providers (both public facilities) felt that water was a concern with regard to IPTp delivery.

### Source, availability of, and concerns about sulphadoxine-pyrimethamine

Public facilities obtained SP supplies free from the state Ministry of Health while the private ones purchased drugs from private suppliers. Twelve public facilities occasionally (but infrequently) had drug stock-outs and used alternative options for obtaining drugs at such times. For instance, four public facilities obtained drugs from private suppliers including patent medicine dealers. They had difficulties getting drugs from the district store since they *“have to source for transport to go and get the drugs at Udi* (local government health department)*”* (provider - rural public facility). On the other hand, though stock-outs were uncommon amongst private providers, most of the private providers reported not waiting until they run out of drugs to make further purchases from the market, in order to avoid interruptions to service delivery, since women had already been billed for ANC in advance. The remaining public facilities and some private providers hand out prescriptions, which women then fill at private outlets and take at home. SP was, however, in stock in all but two facilities (both rural public facilities) on the day of interview. All the providers reported that women generally do not complain of the cost of obtaining SP, and do not seem to be worried about any effect it may have on their babies. Three public and three private providers reported that a few women complained of side effects but this was very infrequent and did not hinder them from taking SP. One added that *“the three SP tablets should be made into one”,* to encourage women to use the drug.

### Availability of staff for delivery of IPTp

Although all private facilities had at least one skilled birth attendant, two out of 11 public facilities in the urban area and seven out of 17 in rural areas did not. In the urban area, most facilities saw at most 10 ANC clients/skilled birth attendant on a given ANC day (Table 3).

**Table 3:** Staff availability for antenatal care

**Variable**	**URBAN**		**RURAL**		**TOTAL**
	**Public**	**Private**	**Public**	**Private**	**34**
	**11**	**3**	**17**	**3**	
At least one skilled attendant available	9	3	10	3	25 (73.5)
No skilled attendant	2	0	7	0	9 (26.5)
ANC client/skilled attendant per day					
1-10	8	1	10	1	20
11-20	0	1	0	1	2
>20	1	1	0	1	3
ANC client/unskilled attendant per day in facility without skilled personnel: 0-5	2	0	7	0	9 (26.5)

The public facility with a client/skilled birth attendant ratio above 20 actually saw 847 women the preceding week, giving an average of 85 ANC clients per skilled birth attendant per day. They had no unskilled staff to support their activities. One private facility in the urban area saw 21 clients/skilled birth attendant per day and had four unskilled staff (auxiliaries) for support services. All the public facilities in the rural area with skilled birth attendants saw at most 10 clients/skilled birth attendant per day. One private facility in the rural area saw 24 clients/skilled birth attendant per day. This facility had six auxiliary staff for support activities. All the facilities without a skilled birth attendant saw at most five women. The staff were mostly CHEWs. With the exception of the provider in the public facility that had the high client/staff ratio noted above, providers at the results dissemination workshop did not think the availability of personnel affects the quality of delivery of ANC services, such as IPTp. Rather, personnel availability was believed to be more important when women were in labour. There was, however, a consensus opinion amongst participants that supervision for ANC activities from senior health officials from within and outside the facility was quite infrequent, and focused mainly on cross-checking facility registers for the number of women enrolled for antenatal care, and the number and outcome of deliveries, but not on the content and process of delivery of most ANC interventions including IPTp.

## Discussion

The finding of this study presents an important insight into existing poor implementation practices affecting IPTp delivery. While most providers were aware of the existence of a national policy on IPTp, their overall knowledge of the guidelines for implementation of the policy was poor. This was more obvious with the knowledge of time of administration of second dose of SP and the use of DOT strategy for IPTp delivery. The overall poor knowledge amongst providers found in this study may have negatively contributed to the low IPTp coverage level of 13.7% and 7.3% for first and second doses, respectively, found for the study area in a related study [6].

The effectiveness of IPTp intervention, which is compromised by the low coverage levels of IPTp in the Nigeria, is further worsened by poor delivery practices for the few women that receive SP. The poor knowledge of guidelines amongst providers was reflected in the poor delivery practices reported amongst providers, whether they were categorized as public/private or across urban/rural. Inappropriate practices such as asking women to take two tablets on the first day, and the third the next day, adjusting the number of tablets to two for women below 50 kg and providing women with SP monthly, have no clinical basis, can compromise effectiveness of SP, and may enhance the development of parasite resistance to SP. The inappropriate adjustment of the number of tablets also means that such women received sub-optimal levels of SP required for the intended protection against malaria. Additionally, the provision of ANC services in some facilities by unskilled staff further raised the likelihood of poor IPTp delivery practices in such facilities.

The practice of DOT is also very poor amongst various categories of providers, and the reasons for such practices raises critical concerns with respect to the effectiveness of IPTp policy. The requirement for women to attend on empty stomach for the purpose of palpation seems to have been institutionalized within some facilities, even though the practice has no scientific basis. A study in Malawi reported that ANC providers felt women should not take drugs on an empty stomach [12]. This is similar to the finding that having come with an empty stomach, women were allowed to take drugs home. The underlying faulty perception among these providers needs to be addressed to enhance the practice of DOT.

It is remarkable that the few providers who had overall correct knowledge did not include a private provider. A study in south-west Nigeria also found poorer knowledge of the guidelines amongst private providers compared to their public sector counterparts, although the respondents were not restricted to those providing antenatal care [14]. Other faulty institutional policies noted among private providers, such as not allowing women to take drugs in the facility, and the assumption that women would take drugs given to them at home, constitute additional impediments to IPTp effectiveness. These findings highlight the importance of observing the knowledge and practices of private providers, and ensuring that they comply with national guidelines.

This study could not verify whether women actually took the drugs that they took home. Nonetheless, observations elsewhere have reported that women may throw such drugs away [19]. Allowing women to take drugs home overrules the very objective of the recommended DOT strategy. Again, not informing women about the drugs they are being given, for whatever reason, conflicts with existing policies for drug administration and safety [20]. It also means that a woman would be unable to report if she had received SP from a provider, and may receive multiple doses if registered with more than one provider.

Unlike the case elsewhere [9,19,21-23], health facility factors such as availability of water and SP in the facility do not seem to be important constraining factors to delivery of IPTp in this setting. Cheap alternative water sources existed, SP stock-outs were uncommon and both providers and the women had insignificant concerns about side effects.

The results of this study therefore support suggestions that provider factors rather than demand-side factors, such as timely attendance to ANC, constrain IPTp delivery in the study area [6].The findings also suggest that interventions targeting improvement in provider knowledge and behaviour, coupled with supportive supervision, should result in improved IPTp delivery. Further research focusing on measuring the effectiveness of provider training and supervision with respect to IPTp delivery is required. In addition, it would be necessary to understand policy-maker and programme-manager behaviour at both state and LGA levels which create the lapse in monitoring implementation of the policy. There is also the need to conduct empirical research using the interpretive policy analysis approach to better diagnose reasons for gaps in the implementation of national IPTp guidelines.

Finally, this paper shows that it is just not enough to have an IPTp policy in place, and attention needs to be paid to how providers implement policy if intended policy objectives of reduction in impact of malaria on pregnant women and their babies are to be achieved. Low coverage of IPTp and use of DOT strategy for its administration is influenced by inappropriate implementation resulting from poor provider knowledge and practices. Policy makers and programme managers of malaria control interventions need to pay attention to monitoring the IPTp provision system in both urban and rural areas as well as amongst public and private providers. Efforts at improving provider practices for IPTp delivery need to go beyond training them, to setting up effective supervisory activities aimed at observing their practices and correcting deviations from recommended policy guidelines. A monitoring system should also be in place to periodically assess the content of antenatal care services being provided by primary providers.

## Competing interests

The authors declare that they have no competing interests.

## Authors’ contributions

CO conceived the study. CO, KH and OO designed the study. CO, OO and BU were involved in data collection and analysis. CO wrote the initial draft of the manuscript and all the authors participated in its finalization.

## References

[B1] FMOH National Antimalaria Treatment Policy 2005 Abuja, Nigeria: National Malaria and Vector Control Division, Federal Ministry of Health

[B2] Falade CO Yusuf BO Fadero FF Mokuolu OA Hamer DH Salako LA Intermittent preventive treatment with sulphadoxine-pyrimethamine is effective in preventing maternal and placental malaria in Ibadan, south-western Nigeria Malar J 2007 6 88 10.1186/1475-2875-6-88 17617910 PMC1941736

[B3] Asa OO Onayade AA Fatusi AO Ijadunola KT Abiona TC Efficacy of intermittent preventive treatment of malaria with sulphadoxine-pyrimethamine in preventing anaemia in pregnancy among Nigerian women Matern Child Health J 2008 12 692 698 10.1007/s10995-008-0319-3 18274885

[B4] Aziken ME Akubuo KK Gharoro EP Efficacy of intermittent preventive treatment with sulfadoxine-pyrimethamine on placental parasitemia in pregnant women in midwestern Nigeria Int J Gynaecol Obstet 2011 112 30 33 10.1016/j.ijgo.2010.07.027 20947080

[B5] National Population Commission and ICF Macro Nigeria Demographic and Health Survey 2008 National Population Commission (Abuja, Nigeria) and ICF Macro 2009 Maryland USA: Claverton

[B6] Onoka CA Hanson K Onwujekwe OE Low coverage of intermittent preventive treatment for malaria in pregnancy in Nigeria: demand-side influences Malar J 2012 11 82 10.1186/1475-2875-11-82 22443266 PMC3364889

[B7] WHO A Strategic Framework for Malaria Prevention and Control during Pregnancy in the Africa Region. In Report AFR/MAL/04/01 2004 Brazzaville: World Health Organization Regional Office for Africa

[B8] WHO Health Services Coverage Statistics: Antenatal Care Coverage (Percentage) 2012 Geneva: World Health Organization

[B9] Hill J Kazembe P Reaching the Abuja target for intermittent preventive treatment of malaria in pregnancy in African women: a review of progress and operational challenges Trop Med Int Health 2006 11 409 418 10.1111/j.1365-3156.2006.01585.x 16553924

[B10] WHO UNICEF Antenatal Care in Developing Countries: promises, achievements and missed opportunities. An analysis of trends, levels and differentials 2003 Geneva: World Health Organization and UNICEF

[B11] Gross K Alba S Schellenberg J Kessy F Mayumana I Obrist B The combined effect of determinants on coverage of intermittent preventive treatment of malaria during pregnancy in the Kilombero Valley Tanzania. Malar J 2011 10 140 10.1186/1475-2875-10-140 PMC3126755 21599999

[B12] Ashwood-Smith H Coombes Y Kaimila M Bokosi M Lungu K Availability and use of sulphadoxine-pyrimethamine (SP) in pregnancy in Blantyre District Malawi Med J 2002 14 8 11 PMC3345411 27528916

[B13] Launiala A Honkasalo ML Ethnographic study of factors influencing compliance to intermittent preventive treatment of malaria during pregnancy among Yao women in rural Malawi Trans R Soc Trop Med Hyg 2007 101 980 989 10.1016/j.trstmh.2007.04.005 17658564

[B14] Fawole AO Onyeaso NC Perception and practice of malaria prophylaxis in pregnancy among primary health care providers in Ibadan, Nigeria West Afr J Med 2008 27 92 96 19025022

[B15] Akinleye SO Falade CO Ajayi IO Knowledge and utilization of intermittent preventive treatment for malaria among pregnant women attending antenatal clinics in primary health care centers in rural southwest, Nigeria: a cross-sectional study BMC Pregnancy Childbirth 2009 9 28 10.1186/1471-2393-9-28 19589164 PMC2719593

[B16] National Bureau of Statistics Federal Republic of Nigeria: 2006 Population Census 2007 Abuja: Federal Government of Nigeria

[B17] Enugu State Ministry of Health Malaria situation in Enugu state 2008 Enugu, Nigeria: Malaria Control Unit,State Ministry of Health

[B18] FMOH Annual Report of the National Malaria and Vector Control Division 2008 Abuja, Nigeria: Federal Ministry of Health 2009

[B19] Mubyazi G Bloch P Kamugisha M Kitua A Ijumba J Intermittent preventive treatment of malaria during pregnancy: a qualitative study of knowledge, attitudes and practices of district health managers, antenatal care staff and pregnant women in Korogwe District North-Eastern Tanzania. Malar J 2005 4 31 10.1186/1475-2875-4-31 PMC1187919 16033639

[B20] FMOH National Drug Policy 2005 Abuja Nigeria: Federal Ministry of Health

[B21] Marchant T Nathan R Jones C Mponda H Bruce J Sedekia Y Schellenberg J Mshinda H Hanson K Individual, facility and policy level influences on national coverage estimates for intermittent preventive treatment of malaria in pregnancy in Tanzania Malar J 2008 7 260 10.1186/1475-2875-7-260 19094198 PMC2630326

[B22] Mubyazi GM Bygbjerg IC Magnussen P Olsen O Byskov J Hansen KS Bloch P Prospects, achievements, challenges and opportunities for scaling-up malaria chemoprevention in pregnancy in Tanzania: the perspective of national level officers Malar J 2008 7 135 10.1186/1475-2875-7-135 18647404 PMC2500039

[B23] Mubyazi GM Bloch P Byskov J Magnussen P Bygbjerg IC Hansen KS Supply-related drivers of staff motivation for providing intermittent preventive treatment of malaria during pregnancy in Tanzania: evidence from two rural districts Malar J 2012 11 48 10.1186/1475-2875-11-48 22340941 PMC3298537

